# Eco-Friendly electrochemical sensor for vildagliptin detection in human plasma: green approach using ZnO nanoparticles and graphene oxide

**DOI:** 10.1186/s13065-025-01582-3

**Published:** 2025-07-18

**Authors:** Bassant Samy, Mokhtar M. Mabrouk, Mohamed A. Abdel Hamid, Hytham M. Ahmed

**Affiliations:** 1 Pharmaceutical Analysis Department, Faculty of Pharmacy, Menoufia University, Shebin Elkom, Menoufia, Egypt; 2 Department of Pharmaceutical Analytical Chemistry, Faculty of Pharmacy, Tanta University, Tanta, El Gharbeia, Egypt; 3 Department of Pharmaceutical Chemistry, Faculty of Pharmacy, Alsalam University, Kafr El Zayat, El Gharbeia, Egypt; 4 Pharmaceutical Analytical Chemistry Department, Faculty of Pharmacy, Menoufia National University, 70 km Cairo-Alexandria Agricultural Road, Menoufia, Egypt

**Keywords:** Modified glassy carbon electrode, Graphene oxide, Zinc oxide nanoparticles, Vildagliptin, Voltammetry, GAPI

## Abstract

A fast, sensitive, and selective voltammetric method utilizing a modified glassy carbon electrode with zinc oxide nanoparticles and graphene oxide (ZnO-NPs / GOs/ GCE) was developed for the determination of vildagliptin (VID). The electrochemical oxidative behavior of VID as well as the morphological characterization of the modified electrode were investigated by transmission electron microscopy, Fourier transform-infrared spectrum and X-ray photoelectron spectroscopy. The determination of VID using the used sensor was shown to have a linear relationship over a concentration range of (15–150 µg/ml) under optimized conditions, with an LOD of 4.9 µg/ml and an LOQ of 15 µg/ml. The used sensor demonstrated good reproducibility, repeatability, and stability. Moreover, the proposed method was successfully used for the determination of VID in pharmaceutical dosage tablets and human plasma with good recovery. The green analytical procedure index (GAPI) method was also used to assess this method’s greenness.

**Supplementary Information:**

The online version contains supplementary material available at 10.1186/s13065-025-01582-3.

## Introduction

Vildagliptin (VID) is an oral antidiabetic agent used to control type II diabetes [1–4]. VID structure (Fig S1) lacks a conjugated double bond, so it doesn’t have specific absorption peaks on the UV spectrum or direct fluorescence. Therefore, the majority of the spectroscopic methods for determination of VID used chemical derivatization with its drawbacks [5–11]. While the other methods, such as HPLC [12–16], and FTIR [17], also used expensive devices, tedious steps and consumed time. In contrast, electrochemical techniques offer a promising alternative due to their low cost, short analysis time, minimal sample preparation, and compatibility with green chemistry principles. On the other hand, VID structure is electrochemically active because it contains an amine group that can be oxidized on an electrode surface. As a result, electrochemical methods have become a focus for developing efficient, accurate, sensitive, and environmentally sustainable analytical techniques for VID determination.

Moreover, VID determination in plasma samples is crucial for therapeutic monitoring of the drug and pharmacokinetic research. These provide additional benefits of employing electrochemical techniques, which eliminate the requirement for extraction or pre-treatment [18–20].

In particular, the application of nanomaterials has significantly enhanced the performance of electrochemical sensors by increasing the surface area, improving electron transfer kinetics, and enabling selective analyte interaction [21–23]. Zinc oxide nanoparticles (ZnO-NPs) are non-toxic, chemically stable, and exhibit excellent catalytic and semiconducting properties [24–26]. Graphene oxide (GO), with its abundant oxygen-containing functional groups and high surface area, offers excellent conductivity and strong adsorption capabilities [27, 28]. When combined, ZnO-NPs and GO can synergistically enhance the electrochemical response of modified electrodes. In this study, we employed a ZnO-NPs/GO composite to modify a glassy carbon electrode (GCE), aiming to enhance sensitivity and selectivity for VID detection. While a variety of materials (e.g., carbon nanotubes, metal-organic frameworks, noble metal nanoparticles) have been investigated for sensor development. ZnO and GO were selected due to their cost-effectiveness, environmental compatibility, and excellent electrochemical performance. Their combination represents a balance between high analytical performance and alignment with green analytical chemistry principles.

The aim of this study is to develop a voltammetric approach for quick as well as accurate estimation of VID in its pure form, pharmaceutical tablets.

Furthermore, the method’s greenness has been evaluated by the Green Analytical Procedure Index (GAPI) [29] in order to achieve our ultimate goal of analyzing VID pharmaceutical formulations with good accuracy and sensitivity while not releasing additional dangerous compounds into the environment. The results revealed that the proposed method was a green method. In recent years, there has been a growing emphasis on developing analytical methods that are not only effective but also environmentally benign. Green analytical chemistry promotes the use of safer solvents, reduced reagent consumption, and low-energy procedures. The proposed electrochemical method aligns well with these principles by employing minimal sample preparation, avoiding toxic organic solvents in the analytical phase, and utilizing a simple, energy-efficient detection system. While a small amount of DMF was used during electrode modification to stabilize the nanomaterial suspensions, its usage was minimized and carefully controlled. Compared to conventional chromatographic techniques, this approach offers a greener, more sustainable alternative for the determination of vildagliptin.

## Experimental

### Instruments

All measurements, including cyclic and differential pulse voltammetry, were performed with the aid of the (OGF500) Potentiostat and the OrigaSoft - OrigaMaster 5 PC software. Voltammetric measurements were performed using a three-electrode system that included ZnO-NPs/GOs/GCE as the working electrode, while the reference electrode was (Ag/AgCl) (sat. KCl), and the counter electrode was platinum (Pt) wire. A digital electronic balance from Switzerland and a digital pH meter (AD-1030) from Adwa have been used. All experiments were conducted at room temperature. Transmission electron microscopy (TEM (was performed using a high-resolution transmission electron microscope (HRTEM, JEOL, Japan) operated at 200 kV accelerating voltage to investigate the morphology of the modified electrode. Fourier transform-infrared spectrum (FTIR) was recorded to identify the surface functional groups of ZnO-NPs/GOs/GCE (Brucker ALPHA II, German). XPS was collected on K-ALPHA (Themo Fisher Scientific, USA) with monochromatic X-ray Al K-alpha radiation − 10 to 1350 e.v spot size 400 micro m at pressure 10 − 9 mbar with full spectrum pass energy 200 e.v and at narrow spectrum 50 e.v.

### Materials and reagents

All experimental solutions were made with analytical grade reagents. The Egyptian Drug Authority (EDA) generously provided VID. VID standard stock solution (300 µg/ml) has been prepared by dissolving 30 mg of VID with 100 ml of deionized water and refrigerating it. VID standard working solution was created through appropriate dilution with stock solution. The commercial tablets (Galvus^®^), each tablet containing 50 mg of VID purchased from a public pharmacy in Cairo, Egypt (Batch No. B8790Y). A 0.1 M phosphate buffer (pH 6–8) was made by combining 0.1 M potassium dihydrogen phosphate, dipotassium hydrogen phosphate, and a suitable amount of NaOH to reach the desired pH [30]. Zinc nitrate hexahydrate, ammonium hydroxide, and polyethylene glycol 4000 (PEG 4000) were purchased from Piochem. Graphene oxide (GO) and DMF were obtained from Sigma-Aldrich (USA).

### ZnO nanoparticle synthesis

A solution consisting of 50 ml of water, 15 ml of polyethylene glycol 4000, and 500 mg of zinc nitrate was prepared. After 30 min of sonication, a 4.00 M solution of ammonium hydroxide was added to adjust the pH to 8.5. Then, the solution was air dried for 30 min. Then the powder was filtered through a 0.45 m filter before being rinsed three times with water and dried at 120 °C. The synthesis was completed by crystallizing the resultant powder for two hours at 65 °C [31].

### Modified working electrode

10 mg of graphene oxide (GO) particles were sonicated in 10 ml of DMF for 2 h to obtain a homogeneous suspension. Also, 10 mg of ZnO nanoparticles were sonicated for 2 h in 10 ml DMF in order to obtain a homogeneous suspension. Prior to surface modification, a glassy carbon electrode (GCE) has been polished with 0.05 μm alumina slurries to achieve a mirror-like surface, which was then treated with ultra-sonication in doubled distilled water. To prepare GO/GCE, 10 µL of GO suspension was drop-coated on the GCE surface and dried at room temperature. The surface was then coated with 10 µL of ZnO nanoparticle suspension and dried to form (ZnO-NPs / GOs/ GCE). The electrode was left to dry at ambient room temperature (25 °C) for approximately half an hour per layer (GO and ZnO).

### General analytical procedures

To characterize the electrochemical behavior of VID and optimize the experimental parameters, cyclic voltammetry was used. Following that, DPV was recorded for quantitative determination. A three-electrode system was immersed in a voltammetric cell containing the required aliquot of VID and completing the volume to 25 ml with phosphate buffer pH 6.5. The optimized operating DPV for sample analysis was as follows: Anodic scanning was used with a potential range of 0.7 to 1.3 V, a scan rate of 25 mV/s, a pulse amplitude of 50 mV, a step potential of 5 mV, and a pulse width of 20 ms.

### Procedure for the commercial tablet

Ten tablets (Galvus^®^) (each containing 50 mg VID) were carefully weighed and grounded in the mortar. An exact weight equivalent to 30 mg VID was added to a 100 ml volumetric flask, containing approximately 50 ml D.W. The flask content was well mixed. The sonication was applied for 30 min, and then the volume was filled with D.W. to the mark. The desired amount of VID was obtained by combining a suitable volume of VID in a voltammetric cell with a phosphate buffer pH 6.5 and analyzing under the same conditions used previously in (Sect. “General analytical procedures”). The regression equation was used to calculate the percent recoveries.

### Spiked human sample procedure

Into a number of centrifugation tubes, 1 ml of plasma is spiked with certain concentrations of VID, 3 ml of methanol (a protein precipitant) is added, and the mixture is then centrifuged at 5000 rpm for about 15 min. The voltametric cell was filled with a suitable amount of phosphate buffer pH 6.5, and 1 ml of centrifuged supernatant was added. The proposed method was then used to analyze the solution.

## Results and discussion

### Characterization of modified electrode

**Fig. 1:**
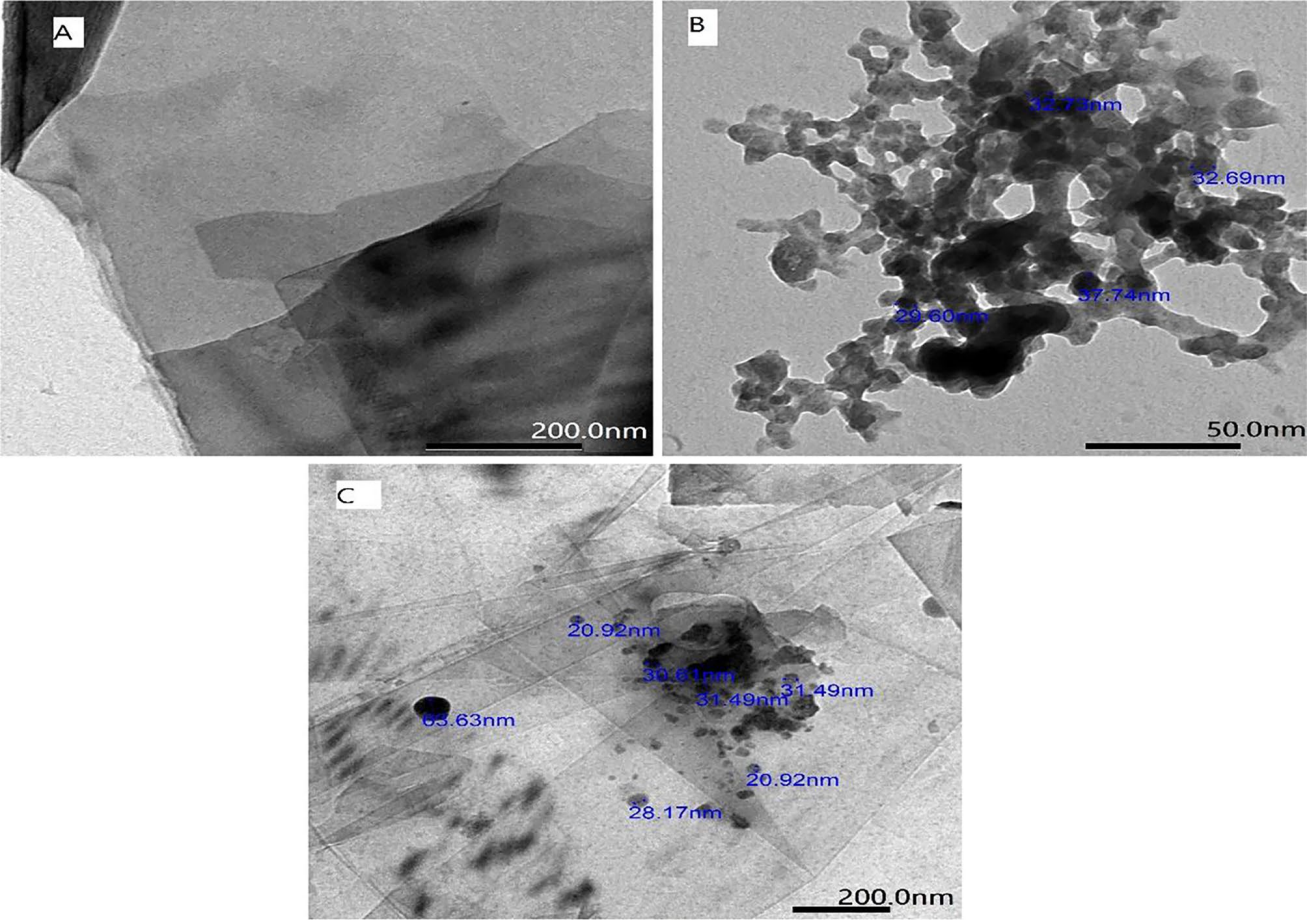
(**A**) TEM images of GO, (**B**) TEM image of ZnO-NPs (**C**) TEM image of ZnO-NPs/GOs

Transmission electron microscopy (TEM) had been used for examination of the morphology and microstructure of the ZnO nanostructure and the ZnO-NPs / GOs nanocomposite modified GCE. The TEM image was examined to confirm the homogeneity of the surfaces and the adhesion of the deposited materials. Figure 1A. is a TEM image of GOs, revealing a sheet-like structure with a high electroactive surface area in comparison with bare GCE. ZnO-NPs are shown as hexagonal nanoparticles in Fig. 1B. Additionally, Fig. 1C. shows ZnO-NPs attached to graphene sheets.

**Fig. 2:**
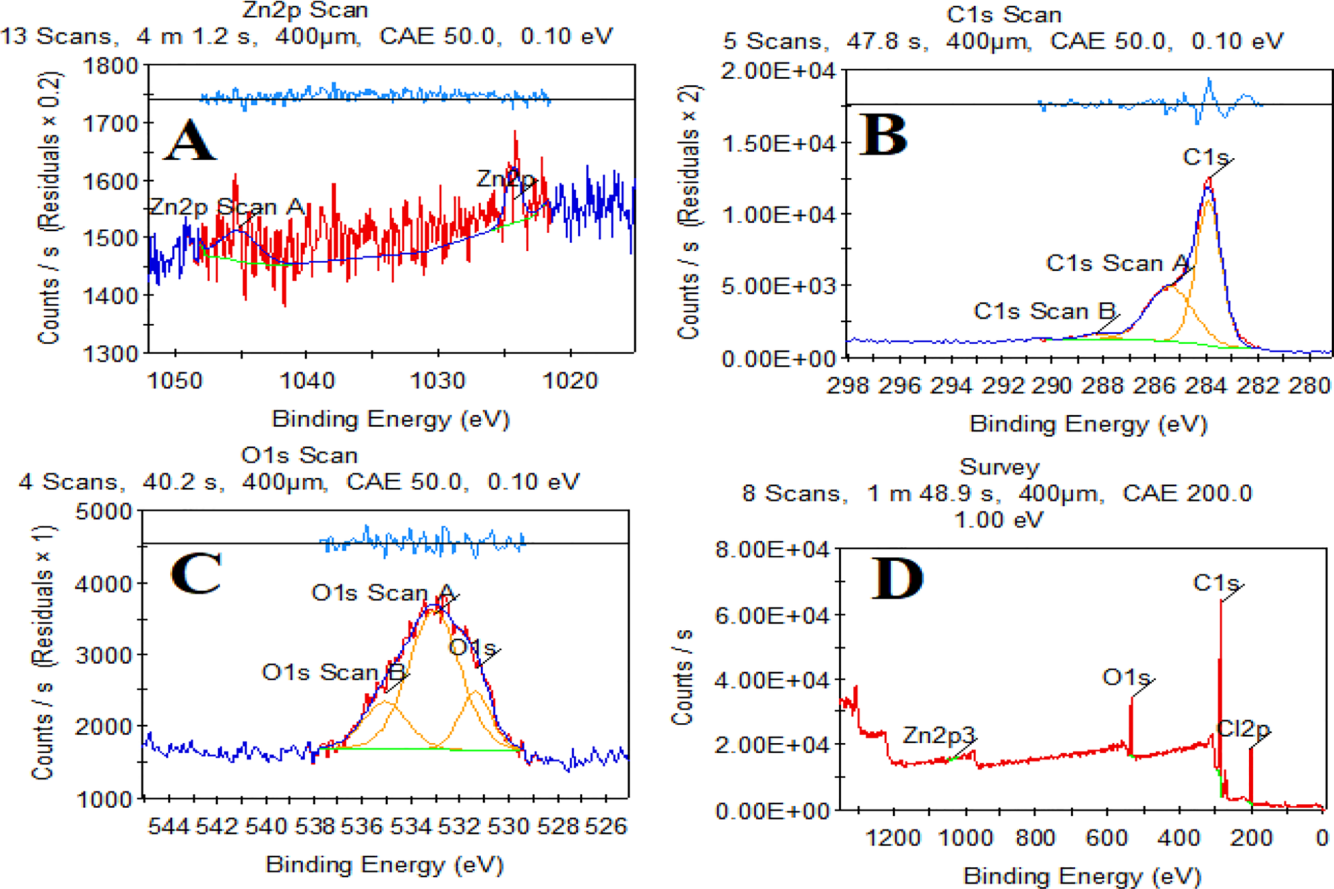
High-resolution XPS spectra of (**A**) Zn 2p, (**B**) O 1s, and (**C**) C 1s regions for the ZnO-NPs/GOs/GCE composite. (**D**) Survey scan confirming elemental composition

X-ray photoelectron spectroscopy (XPS) analysis confirmed the chemical composition of the modified glassy carbon electrode (GCE) decorated with zinc oxide nanoparticles and graphene oxide (ZnO-NPs/GOs/GCE)in Fig. 2. The survey spectrum revealed dominant peaks corresponding to zinc (Zn), oxygen (O), and carbon (C), with no detectable impurities Fig. 2D.

High-resolution scans of the Zn 2p region exhibited two symmetric peaks at binding energies of approximately 1022 eV and 1045 eV, separated by a spin-orbit splitting of 23 eV. This doublet is characteristic of structure of ZnO, confirming the successful immobilization of ZnO nanoparticles on the electrode surface Fig. 2A.

The O 1s spectrum was deconvoluted into three components. The primary peak at 530 eV corresponds to lattice oxygen in ZnO, while the secondary peak at 531.5 eV is attributed to surface hydroxyl groups or adsorbed water. A third component at 533 eV arises from oxygen-containing functional groups in graphene oxide Fig. 2C.

Deconvolution of the C 1s spectrum revealed four distinct contributions. The main peak at 284.5 eV is assigned to hybridized carbon (C-C/C = C) from the graphene oxide and the underlying GCE Fig. 2B.

The XPS results collectively demonstrate the coexistence of ZnO nanoparticles and graphene oxide on the GCE surface, with chemical states consistent with their expected structures.

**Fig. 3:**
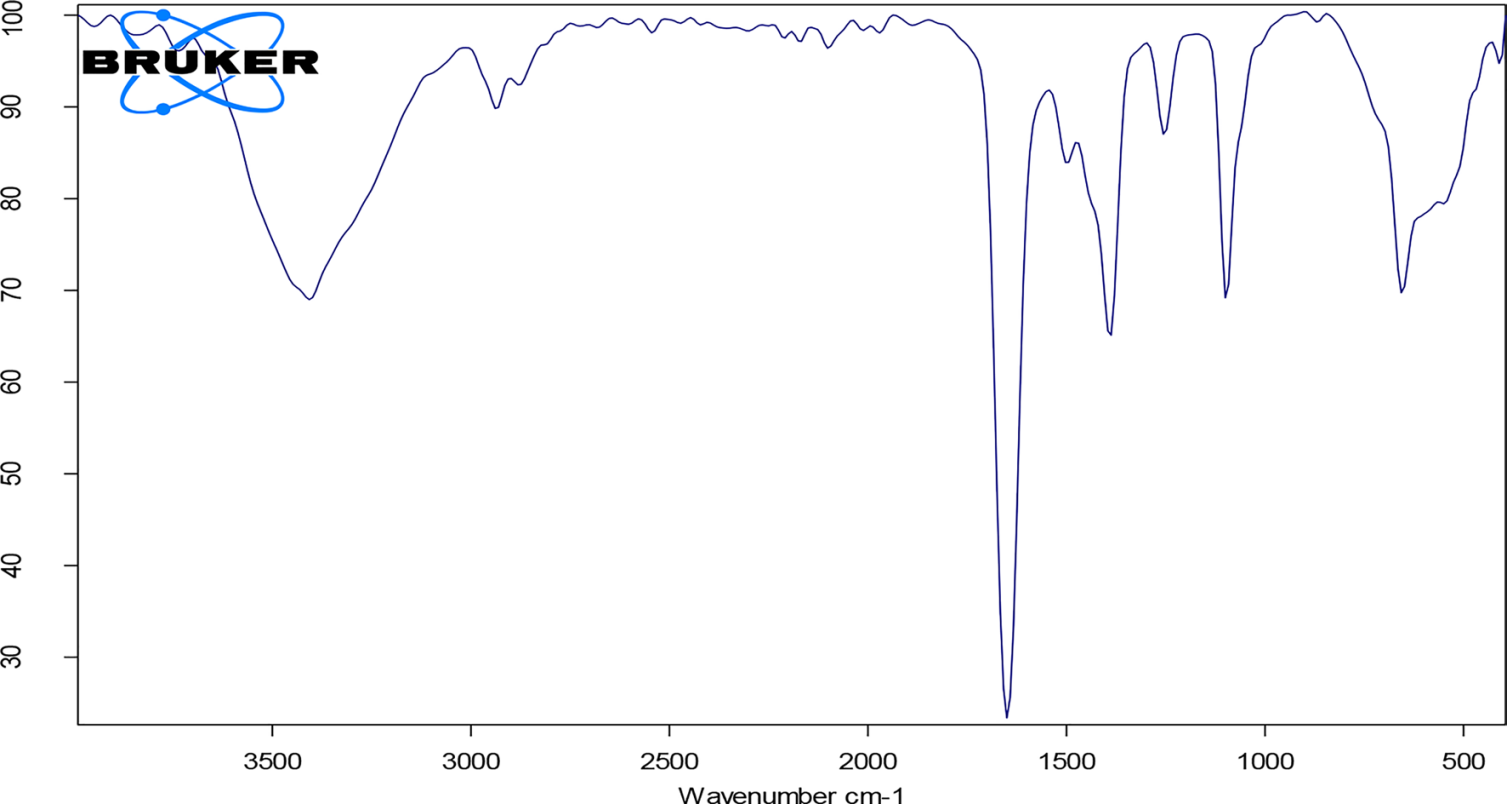
FTIR spectrum of the ZnO-NPs/GO modified glassy carbon electrode, confirming the presence of functional groups associated with graphene oxide and ZnO nanoparticles

The FTIR spectrum of the ZnO-NPs/GOs modified glassy carbon electrode confirmed the presence of characteristic functional groups related to both graphene oxide and ZnO nanoparticles. A broad band observed between 3400 and 3200 cm⁻¹ corresponds to O–H stretching vibrations, which attributed to hydroxyl groups present in graphene oxide. The peak in the range of 1720 to 1700 cm⁻¹ is associated with C = O stretching, indicating the presence of carboxylic acid groups resulting from oxidation of the graphene framework. The absorption band around 1620 to 1580 cm⁻¹ is related to C = C stretching vibrations, which are typical for the sp² carbon skeletal structure of graphene. A distinct absorption band in the range of 500 to 400 cm⁻¹ corresponds to Zn–O stretching vibrations, confirming the incorporation of ZnO nanoparticles. These spectral features collectively verify the successful surface modification of the electrode with graphene oxide and zinc oxide components Fig. 3.

### Electrochemical behavior of VID

**Fig. 4:**
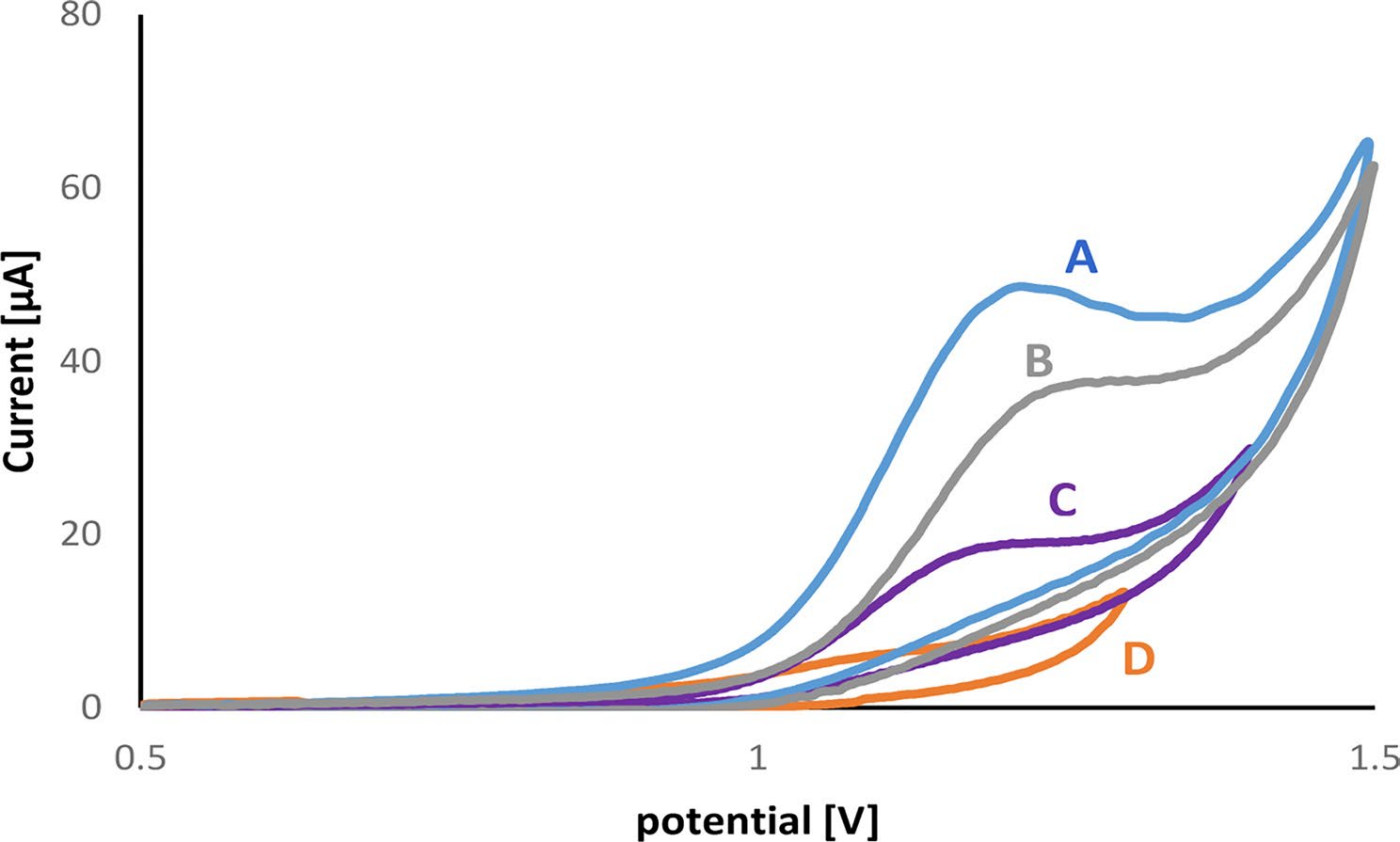
(**A**) Cyclic voltammogram (CV) of 1.0 × 10^− 3^ M VID at ZnO-NPs/GOs/GCE sensor in phosphate buffer buffer at pH 6.5 and scan rate 100 mV /s (**B**) CV of 1.0 × 10^− 3^ M VID at ZnO-GO dispersion at GCE sensor in phosphate buffer buffer at pH 6.5 (**C**) CV of 1.0 × 10^− 3^ M VID at glassy carbon electrode (**D**) Blank

A cyclic voltammogram of 1 mM VID in phosphate buffer of pH 6.5 was measured on the ZnO-NPs/GOs/GCE across the potential range between 0.5 and + 1.4 V (Fig. 4). A figure illustrates that VID had one irreversible oxidation peak without a reduction peak. The peak anodic current observed with the ZnO-NPs / GOs / GCE electrode (Fig. 4A) was greater than that of the unmodified glassy carbon electrode (Fig. 4C). The enhanced catalytic activity and active surface area of the modified electrode led to a significant improvement in its conductivity and current sensitivity. The figure also shows the anodic peak potential (+ 1.16 V) and current (50.9 µA) when compared with the previously reported method, in which VID had been oxidized using the CV approach at Pt and GC electrodes with potential ranges between + 1.25/+1.45 V and + 1.15/+1.25 V, respectively [32]. According to those findings, the electrochemical process occurring at the modified electrode (ZnO-NPs/GOs/GCE) is easier than that occurring at GC/Pt electrodes. For optimal electrochemical performance, graphene oxide (GO) was first deposited onto the GCE surface, followed by ZnO nanoparticle deposition. This sequential modification ensured better surface coverage, more stable film formation, and improved electron transfer. As shown in Fig. 4A, this method of electrode preparation resulted in higher sensitivity and peak current compared to the electrode modified with pre-mixed ZnO-GO dispersions (Fig. 4B). Figure 4D displays the cyclic voltammogram of the blank (phosphate buffer, pH 6.5) without VID, confirming the absence of any interfering oxidation peaks from the background electrolyte.

### Electroactive surface area

The electroactive surface area of the modified glassy carbon electrode (ZnO/GO/GCE) was evaluated using cyclic voltammetry with 0.5 mM potassium ferricyanide in 0.1 M KCl as the supporting electrolyte. Cyclic voltammograms were recorded at scan rates ranging from 30 to 130 mV/s (Fig. S2). A well-defined pair of redox peaks was observed, and the anodic peak current (Ip) increased linearly with the square root of the scan rate (*V*^1\2^). The electroactive surface area (A) was calculated using the Randles–Ševčík equation for a reversible system at room temperature (25 °C) [33]:

$${\rm{Ip}}{\rm{ = (2}}{\rm{.69 \times 1}}{{\rm{0}}^{\rm{5}}}{\rm{) \times }}{{\rm{n}}^{{\rm{3/2}}}}{\rm{ \times A \times }}{{\rm{D}}^{{\rm{1/2}}}}{\rm{ \times C \times }}{{\rm{v}}^{{\rm{1/2}}}}$$

Where Ip ​ is the peak current (A), n is the number of electrons transferred (*n* = 1), A is the electroactive surface area (cm²), D is the diffusion coefficient of potassium ferricyanide (7.6 × 10⁻⁶ cm²/s), C is the concentration (mol/cm³),V^1\2^ is the square root scan rate (V/s). The plot of Ip​ versus ν^1\2^yielded a straight line with slop equal 28.12µA·(V/s)^-1\2^. Substituting the slope into the Randles–Ševčík equation, the electroactive surface area of the ZnO/GO/GCE was calculated to be 0.076 cm². This is higher than that of the bare GCE (0.069 cm^2^) highlighting the enhancement in active surface area and electron transfer kinetics due to the synergistic effect of ZnO nanoparticles and graphene oxide.

### Optimization of method’s variable

#### Assessing effect of scan rate

**Fig. 5:**
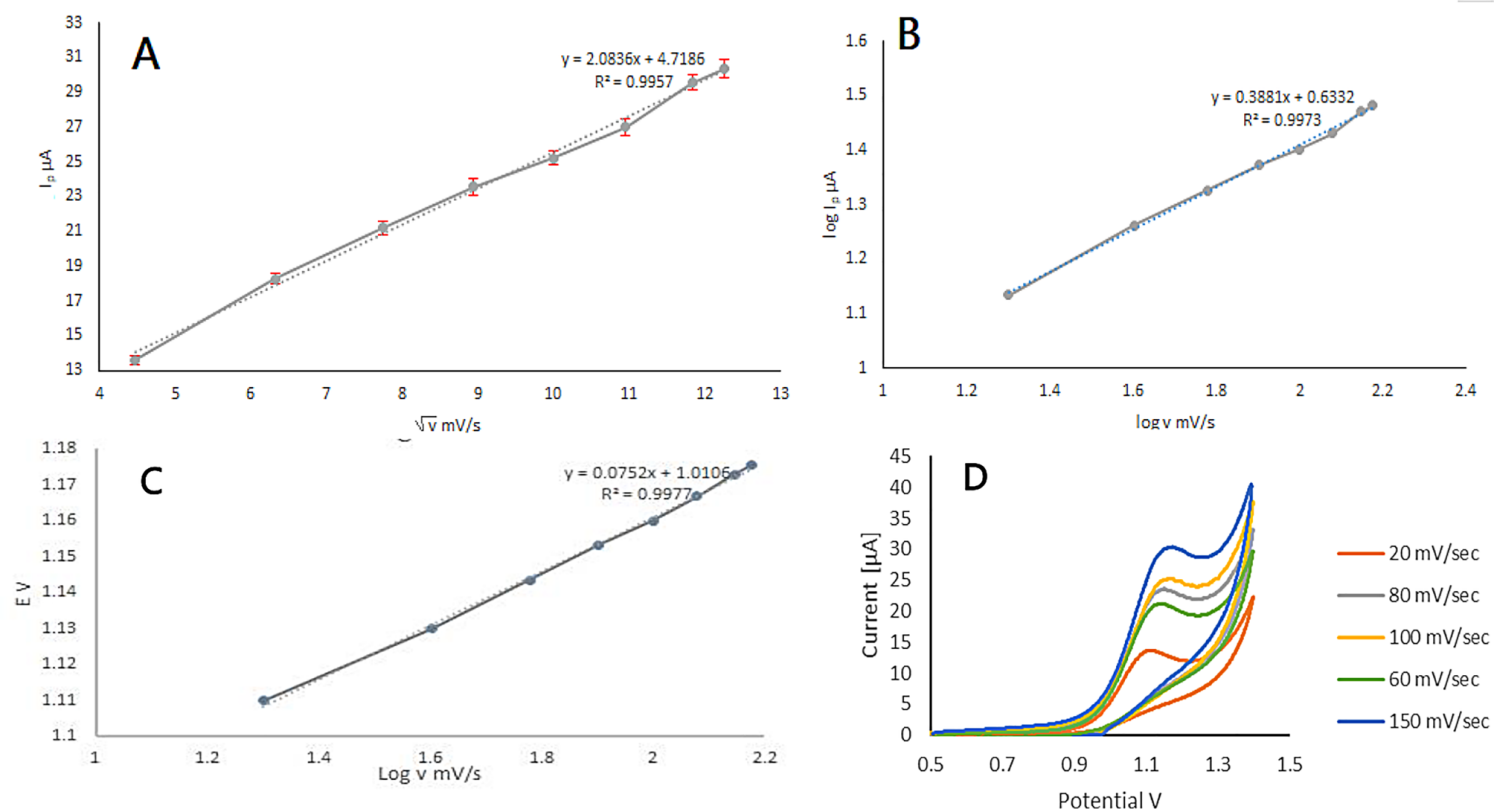
Effect of √ scan rate υ on the peak current I_p_ (mean ± SD, *n* = 3) (**A**), log υ on log I_p_ (**B**) and log υ on the potential E (**C**) of VID drug at ZNO-NPs/GOs/GCE sensor at pH 6.5 (**D**) cyclic voltammetry of 0.5 mM VID in phosphate buffer of pH 6.5 medium performed at various potential scan rates (20–150 mV/s)

To investigate the effect of potential scan rate (υ) on anodic peak signal, cyclic voltammetry of 0.5 mM VID in phosphate buffer of pH 6.5 medium was performed at various potential scan rates (20–150 mV/s) Fig. 5D. shows that the data related to the relationship between the scan rate (√υ) and current peak (Ip) revealed a linear relationship with *r* = 0.995 (Ip (µA) = 2.08 √υ (mV/s) + 4.7) (Fig. 5A). Also, plotting Log υ and Log Ip yielded a straight line with *r* = 0.997 (Log Ip = 0.388 Log υ + 0.633). The slope was 0.388, which is close to the theoretical value of 0.5 (Fig. 5B). According to this data, it demonstrates that this electrochemical oxidation that occurred in phosphate buffer of pH 6.5 medium can be diffusion controlled using a modified electrode.

Moreover, the anodic peak potential turns to higher positive values as the scan rate rises. This phenomenon is expected to be characteristic of irreversible reactions [34]. The relationship between potential (Ep) and scan rate (υ) is defined as Ep(V) = 0.075 Log υ (mV/s) + 1.01, *r* = 0.997. The laviron equation, illustrated below, can be utilized for calculating the electron number involved in the electrochemical reaction [35].

E = Eº + ($\:\frac{2.303\:RT}{{\upalpha\:}\text{n}\text{F}})$log ($\:\frac{RTK}{{\upalpha\:}\text{n}\text{F}})$+$\:\:\left(\frac{2.303\:RT}{{\upalpha\:}\text{n}\text{F}}\right)$log υ

When plotting log scan rate versus potential Ep (V) = 0.075 log υ (mV/s) + 1.01, the resulted slop can be used to calculate αn. (Fig. 5C) Where n is the number of electrons transferred and α is the electron transfer coefficient.

**Scheme. 1:**
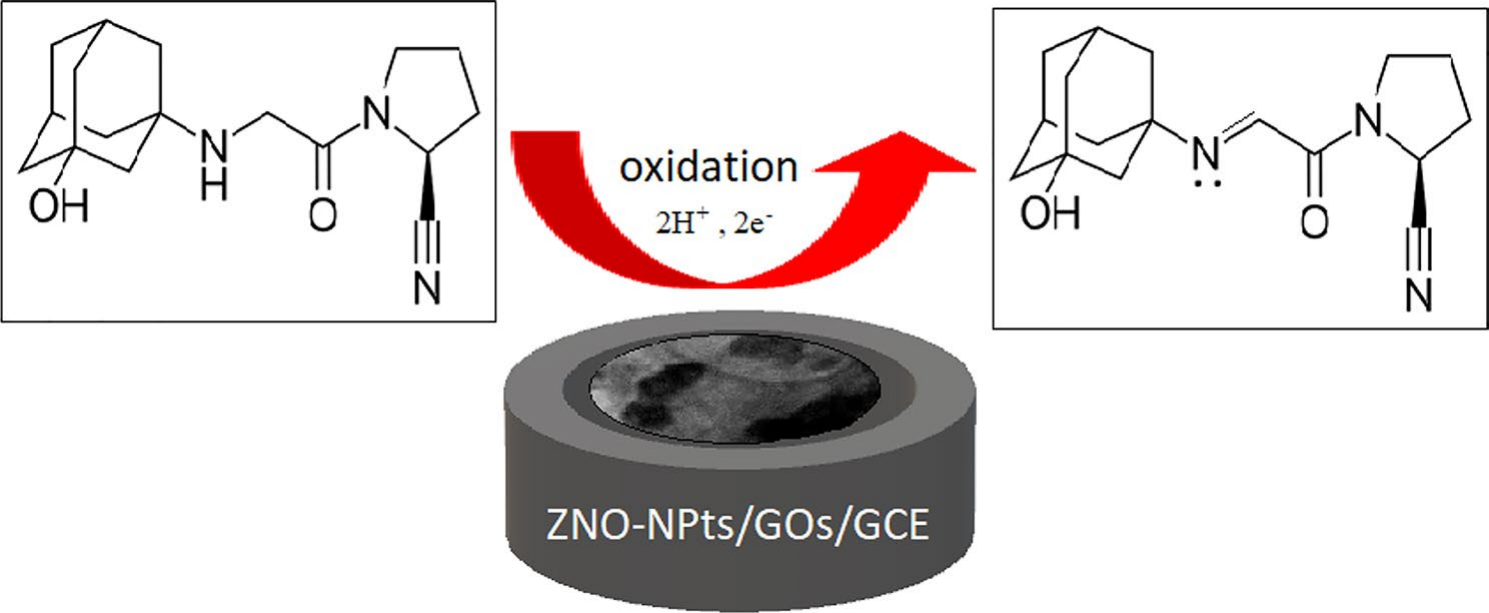
Proposed oxidation mechanism of VID

The slope is 0.075, according to the previous equation. Since VID electro-oxidation had been irreversible, α was determined to be 0.5 and n to be 1.76 (≈ 2), which matched the mechanism proposed in Scheme 1.

Based on the observed 2-electron transfer process, the electrochemical oxidation of vildagliptin (VID) is believed to proceed via the following pathway:

The mechanism begins with the removal of an acidic α-proton adjacent to the α-carbon of the carbonyl group, resulting in the formation of a carbanion intermediate. This carbanion undergoes one-electron oxidation to generate a carbon-centered radical. Subsequently, intramolecular interaction of this radical with the adjacent nitrogen atom leads to the formation of a nitrogen-centered radical intermediate. A second one-electron oxidation step yields a quaternary ammonium-type Schiff base intermediate, which upon proton loss, forms an imine VID [36, 37].

This mechanism involves a total of two electrons and two protons, which aligns well with the electrochemical findings from Laviron analysis and supports the irreversible nature of the process observed in the voltammetric experiments. The overall oxidation pathway is shown schematically in Scheme 1.

#### Assessing effect of pH

**Fig. 6:**
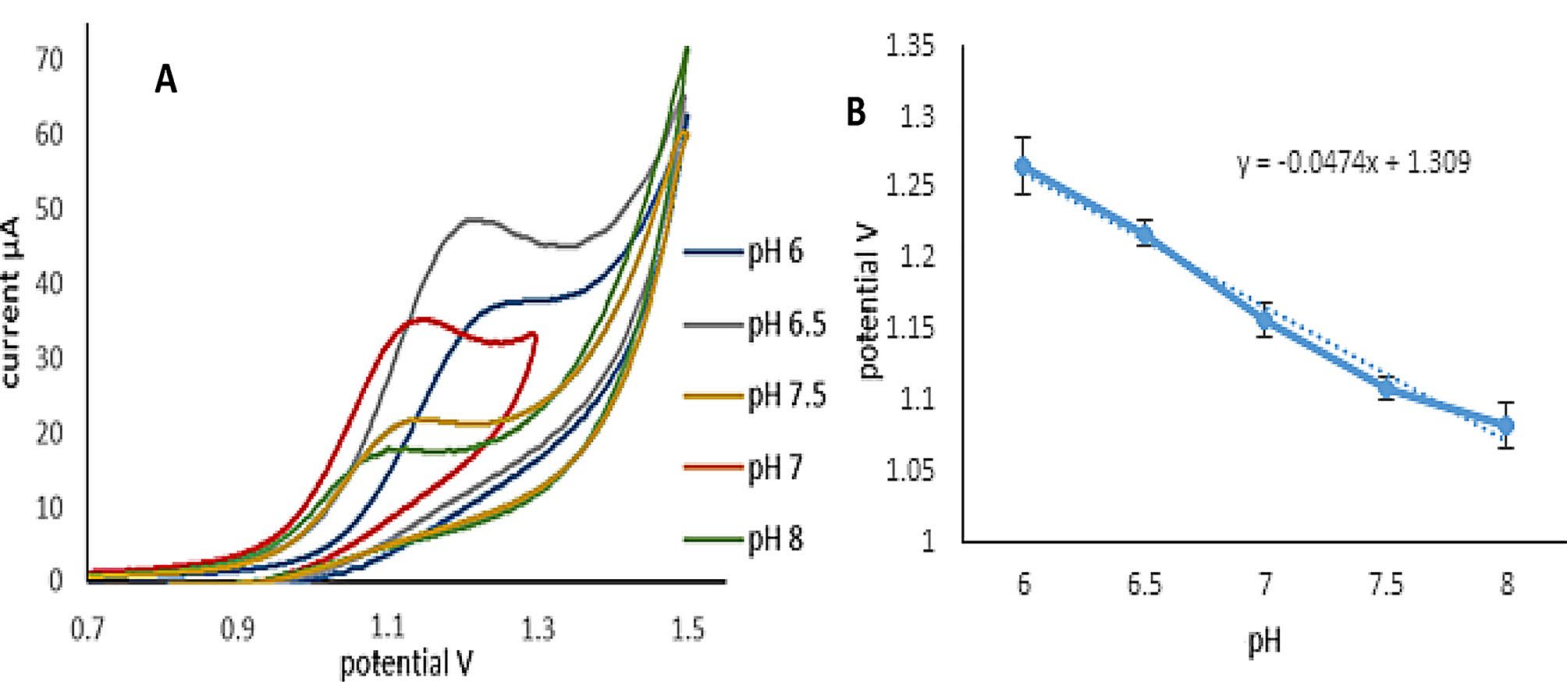
Effect of pH on the peak current (**A**) and peak potential (mean ± SD, *n* = 3) (**B**) of VID in phosphate buffer using the ZnO-NPs/GOs/GCE sensor

The influence of pH on the electrochemical analysis of VID was investigated using phosphate buffer with pH varying from 6 to 8. The pH of a solution was discovered to alter electrochemical characteristics such as peak current and VID oxidation potential. The peak current (Ip) vs. pH plot revealed that pH 6.5 had the highest peak current value; thus, this pH was chosen as the ideal pH, and the relationship between peak potentials and the solution pH of VID was investigated as well (Fig. 6.). The peak potential was shown to change negatively when pH values increased, demonstrating the involvement of protons in the oxidation reaction. The linear relationship between peak potential and solution pH was described by the following equation: E(V) = − 0.047 pH + 1.309 The slope was calculated to be (0.047), which is close to the Nernst equation’s value and implies that the number of protons and electrons involved in the electrode reaction is the same [38]. 

### Validation of the proposed method

The ICH guidelines had been followed in this work for validation of the proposed method [39]. The validation parameters include linearity range, precision and accuracy, detection, and quantitation limits.

#### Linearity range

**Fig. 7:**
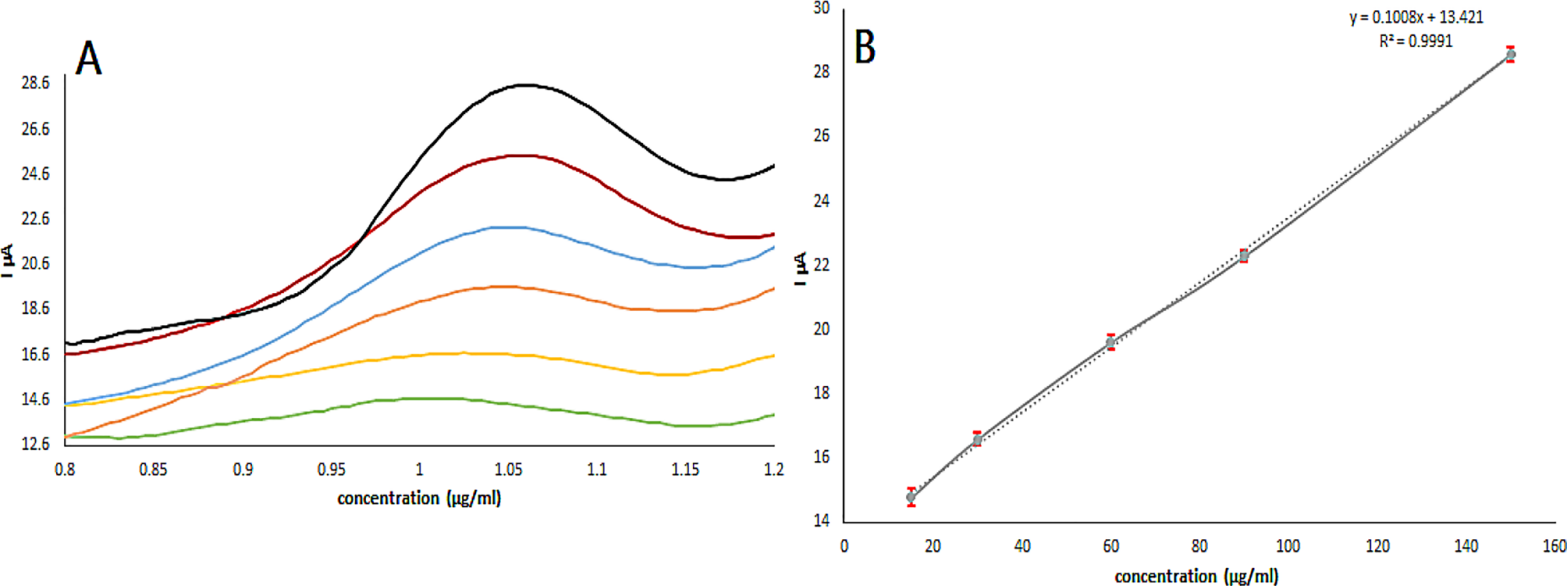
DPV curves (**A**), corresponding calibration curve (mean ± SD (*n* = 3)) (**B**) recorded for different concentrations of VID (15–150 µg/ml) at ZNO-NPs/GOs/GCE sensor in phosphate buffer pH 6.5 and scan rate 100 mV/s

Figure 7 exhibits the DPV response of ZnO-NPs/GOs/GCE to VID in the concentrations that range from 5 to 150 µg/ml, and their linear calibration curve was obtained within the mentioned concentration range (Fig. 7B). The calibration curve showed a slightly positive shift in the peak potential at higher concentrations. Because the diffusion process is interrupted at high concentrations of analyte, diffusion occurs satisfactorily at low concentrations. Therefore, in order to overcome this issue, the electrochemical method uses extra potential. It is very probable that VID deposition on the electrode surfaces was the cause of the linearity loss above these values. The statistical analysis of the data gave a high value for the correlation coefficient (r), as presented in Table 1.

#### Limits of detection (LOD) and quantitation (LOQ)

The proposed electrochemical method sensitivity was evaluated using the limits of detection (LOD) and quantification (LOQ) values. They were calculated with the following formula: LOD = 3.3 σ / S, LOQ = 10 σ /S, where S is the calibration curve slope and σ is the standard deviation of the intercept [40]. The LOD and LOQ values were discovered to be 4.9 and 15 µg/ml, respectively. Which indicates the high sensitivity of the proposed approach. These limits are higher than typical therapeutic plasma concentrations. This reflects that the method is primarily optimized for quality control and pharmaceutical formulation analysis, where analyte concentrations are generally higher. The method is also well-suited for pharmacokinetic studies involving sample pre-concentration, allowing accurate measurement in concentrated plasma samples. Moreover, compared to previously reported methods [32, 41–43]for the determination of vildagliptin in dosage forms, our method demonstrates superior limits of quantification (LOQ), greenness, or cost-effectiveness, as shown in Table 2, confirming its suitability for pharmaceutical formulation analysis.

**Table 1:** Regression and quantitative parameters for VID determination by ZnO-NPs/GOs/GCE for the proposed method

Parameter	VID
Linearity Range (µg/ml)	15–150
Intercept (a)	13.42
SD of Intercept (S_a_)	0.14
Slope (b)	0.1
SD of Slope (S_b_)	0.001
Correlation coefficient (r)	0.9995
Coefficient of determination (r^2^)	0.999
SD of residual (S_y/x_)	0.19
Accuracy^a^ (Mean ± SD)	99.88 ± 0.1
Limit of detection (µg /mL)	4.9
Limit of quantitation (µg /mL)	15
Intra-day of peak current (RSD%)	0.23
Inter-day of peak current (RSD%)	0.29

^a^ mean of five determinations

**Table 2:** Comparative evaluation of analytical methods for VID determination in dosage forms

Methods	LOD (µg/mL)	LOQ (µg/mL)	Cost	Greenness	References
Modified ZNO-NPs/GOs/GCE	4.8	15	Not expensive	Green	This method
HPLC	4.45	10.15	Expensive instrument	Using organic solvent such as acetonitrile	[38]
HPTLC	1021.25 ng/band	3094.70 ng/band	Expensive instrument	Using organic solvent such as Toluene and Ethyl acetate:	[38]
HPLC	2.98	9.94	Expensive instrument	Using organic solvent such as acetonitrile and methanol	[39]
Voltammetric method used GCE	73.5094	243.327	Not expensive	Green	[29]
Spectrophotometry	5.1	15.5	Moderate with reaction time 20 min	Using acetonitrile solvent	[40]
Spectrophotometry	5.17	15.66	moderate	Using DMF as solvent	[40]

#### Accuracy

The estimation of VID in pure form using DPV yielded good recovery results (Table S1). To estimate the accuracy of the proposed method, five VID concentrations within the linear range (15–150 µg/ml) were tested. In addition, three replicates were examined for each concentration, and the results were presented as (% recovery ± RSD) in Table S1. The obtained data demonstrated that the current method is accurate.

#### Precision and stability

The repeatability and intermediate precision of the proposed method were assessed by the analysis of three different VID concentrations on one day (intraday precision) and three consecutive days (interday precision). The proposed method results were evaluated and demonstrated high precision, as indicated by lower values of %RSD. (Table S2).

The long-term stability of modified glassy carbon electrode (ZnO-NPs / GOs/ GCE) was investigated by measuring the current response at a fixed VID concentration of 50 µg/ml over a period of a seven days. The modified electrode was used daily and stored in room temperature. The experimental conditions show that the current response only deviates by 4.4% over a week which show good stability. Fig S3 show % Percentage deviation of the current response of the modified electrode over 7 days during repeated measurements of vildagliptin, demonstrating long-term stability.

Additionally, the reusability of the modified electrode was evaluated by conducting 20 consecutive measurements of vildagliptin. The results demonstrated consistent current responses with no significant signal loss, indicating excellent electrode durability and reusability.

Electrode-to-electrode variability was evaluated using five independently fabricated ZnO-NPs/GOs/GCE electrodes. Each electrode was used to measure the oxidation peak current of 80 µg/ml VID under the same experimental conditions. The relative standard deviation (%RSD) was calculated to be **1.99%**, demonstrating excellent reproducibility of the electrode fabrication process (Table S2).

#### Selectivity

To check up on the interference effect, the selectivity of the adopted procedure was tested by calculating %recovery obtained from the analysis of VID tablets. It was discovered by analyzing VID by the proposed method that there is no interference from excipients found in pharmaceutical preparations such as lactose, anhydrous cellulose, microcrystalline sodium starch glycolate (type A), and magnesium stearate. Based on a mean of five determinations, the mean percentage recoveries and SD values obtained were 100.2 ± 0.38. As a result, the proposed method demonstrated high selectivity (Table 3**).**

**Table 3:** Application of the proposed DPV method for assay of VID in tablet dosage form

Parameter	Galvus^®^ tablets	
	Proposed method	Reported method [39]
% Mean Recovery	100.2	100.02
Standard deviation (SD)	0.38	0.35
Number of determinations	5	5
t-Value^a^	0.43	
f-Value^a^	5.2	

^**a**^ tabulated value at 95% confidence limit; t = 2.306 and F = 6.338

Since metal oxide nanoparticles (ZnO) have a remarkable catalytic activity and adsorption capacity due to their sizes and shapes, they are frequently used for the detection of pharmaceutical analytes [44–46]. Graphene oxide (GO) is a layered material formed by the oxidation of graphite. Furthermore, it has a large surface area and excellent electrical conductivity, which make it an excellent candidate for use as electrochemical sensors. The synergism of metal-carbon nanotechnology has been extensively used for sensing applications because of their large surface area, excellent conductivity, and affordable price [47–49].

## Applications

### Analysis of VID in its tablets

The efficiency of the proposed procedure for analyzing VID in commercial tablets was investigated. The DPV at (ZnO-NPs/GOs/GCE) sensor was used to estimate VID in Galvus^®^ tablets. The t-test and F-test were used to compare the statistical results of the suggested approach with those of the reported method, and the results showed no discernible difference between both of them [11]. The suggested approach displayed an excellent recovery (Table 3).

### VID assay in spiked human plasma

The suggested approach was successfully used to estimate VID in spiked human plasma. VID is absorbed quickly, taking 1.5 to 2 h to reach a peak plasma concentration level. Table S3 displays the results and percentage recoveries of standard VID solutions (15,20, 50, 80, and 120) µg/ml added to 1 ml drug-free plasma, mixed, and analyzed as described in Experimental Sect. “Spiked human sample procedure”. For each concentration, three independent measurements were made. The percentage of VID recovered in spiked plasma samples ranged between 98.38 and 101.6%. These results demonstrated the reliability of the mentioned technique and its capacity to determine VID in spiked plasma. This method did not result in any interference with the oxidation peak from other additives (Fig S2).

### Greenness assessment

**Fig. 8:**
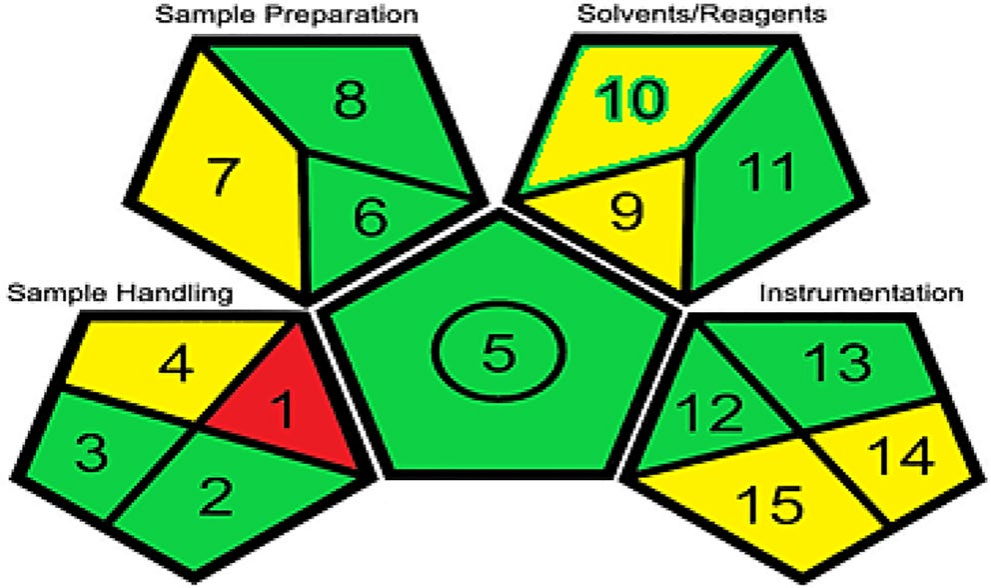
Green Analytical Procedure Pictograms for proposed method

The proposed method’s greenness profile was evaluated using analytical GAPI tools. The recent greenness evaluation technique, GAPI, is a visual presentation method comprised of five pentagrams. These pentagrams assess the environmental impact of the method’s main steps (sample collection, preservative, transportation and storage, sample preparation, reagents, and instrumentation) using red, yellow, and green colors high, medium, and low hazardous effects, respectively [50]. The suggested method, GAPI pentagrams (Fig. 8.), exhibits a green color dominance, indicating a low harmful environmental effect.

While electrochemical methods are often considered green due to their inherent properties, this study employed the GAPI tool to objectively assess the method’s environmental impact, confirming a favorable profile with minimal use of toxic solvents and low waste generation. Compared to conventional methods for vildagliptin determination—such as HPLC or spectrophotometric assays—the proposed method avoids the use of large volumes of organic solvents, minimizes hazardous waste generation, and employs relatively low-energy instrumentation. In contrast, traditional HPLC methods often involve toxic solvents (e.g., acetonitrile, methanol), longer run times, and higher energy consumption which demonstrated at Table 2. Therefore, the current method offers a more sustainable and environmentally benign alternative for routine analysis.

## Conclusion

A DPV method had been established and successfully implemented or the determination of VID utilizing the ZnO-NPs/GOs/GCE sensor in pure form, pharmaceutical dosage tablets, and spiked plasma. The electrode modification gives a quick response time, low detection limits, and is cost-effective. This study also provided further information regarding the electrochemical characteristics of VID. This voltammetric method demonstrated excellent linearity in the 5–150 µg/ml range, with an analytical signal obtained at phosphate buffer pH 6.5. Based on the validation parameters, the suggested approach demonstrated remarkable precision, specificity, and accuracy. Additionally, according to the GAPI tool, the suggested electrochemical method demonstrated a favorable greenness profile, as confirmed by GAPI assessment.

## Electronic supplementary material

Below is the link to the electronic supplementary material.

Supplementary Material 1

## Data Availability

The data that support the findings of this study are available within the manuscript and its supplementary information files. Additional raw data related to electrochemical measurements and characterization are available from the corresponding author upon reasonable request.

## Abbreviations

GCE: Glassy Carbon Electrode
ZnO-NPs: Zinc Oxide Nanoparticles
GO: Graphene Oxide
CV: Cyclic Voltammetry
LOD: Limit of Detection
LOQ: Limit of Quantification
RSD: Relative Standard Deviation
VID: Vildagliptin
DMF: N, N-Dimethylformamide
HPLC: High Performance Liquid Chromatography
HPTLC: High-Performance Thin-Layer Chromatography
µg/mL: Microgram per milliliter
mV/s: Millivolt per second
GAPI: The green analytical procedure index
TEM: Transmission electron microscopy
FTIR: Fourier transform-infrared spectrum
XPS: X-ray photoelectron spectroscopy
