# Supplementary material for: Eco-Friendly electrochemical sensor for vildagliptin detection in human plasma: green approach using ZnO nanoparticles and graphene oxide
Source: BMC Chem. 2025 Jul 18;19(1):216. doi: 10.1186/s13065-025-01582-3 (PMC12273015; doi:10.1186/s13065-025-01582-3)
Supplement: Supplementary file 1 — Supplementary Material 1 [file 13065_2025_1582_MOESM1_ESM.docx]

**Eco-Friendly Electrochemical Sensor for Vildagliptin Detection in human plasma: Green Approach Using ZnO Nanoparticles and Graphene Oxide**

**Bassant Samy ^1^ ,Mokhtar M. Mabrouk ^2,3^, Mohamed A. Abdel Hamid ^2,3^, Hytham M. Ahmed ^1,4^**

^1^Pharmaceutical Analysis Department, Faculty of Pharmacy, Menoufia University, Shebin Elkom, Menoufia, Egypt.

^2^Department of Pharmaceutical Analytical Chemistry, Faculty of Pharmacy, Tanta University, Tanta, El Gharbeia, Egypt.

^3^Department of Pharmaceutical Chemistry, Faculty of Pharmacy, Alsalam University, Kafr El Zayat, El Gharbeia, Egypt.

^4^Pharmaceutical Analytical Chemistry Department, Faculty of Pharmacy, Menoufia National University, 70 km Cairo-Alexandria agricultural road, Menoufia, Egypt.

Corresponding author: Tel: 00201004844589

E-mail: [hmaahmed@yahoo.co.uk](mailto:hmaahmed@yahoo.co.uk)

**
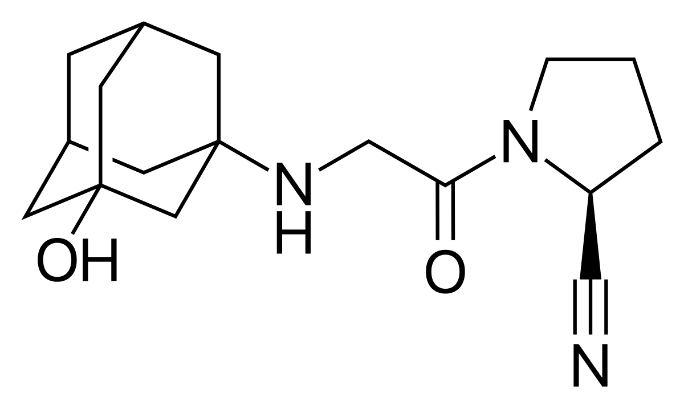
**

**Fig S1.** Chemical structure of Vildagliptin.

**
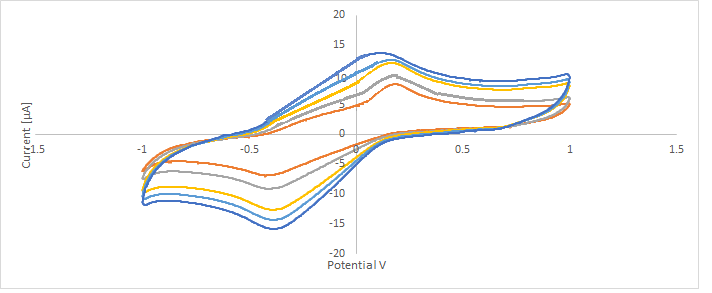
**

**Fig S2.** Cyclic voltammograms of 0.5 mM [Fe(CN)^6^]^3−/4-^ in 0.1 M KCl at ZnO/GO/GCE at various scan rates (30–130 mV/s)


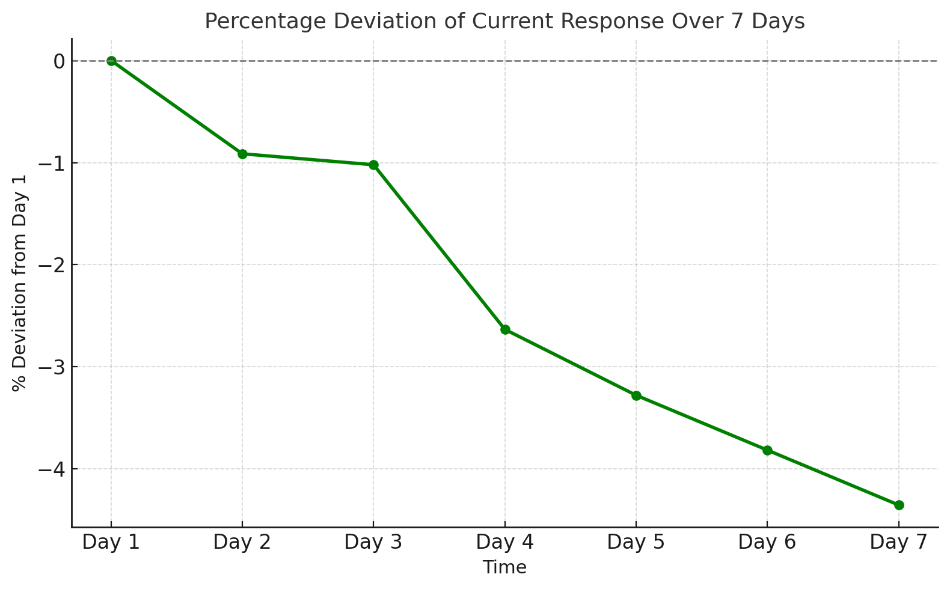


**Fig S3.** Percentage deviation of the current response of the modified electrode over 7 days during repeated measurements of vildagliptin, demonstrating long-term stability

**Fig S4.** DPV of 20μg/ml of VID in spiked plasma at ZNO-NPs/GOs/GCE sensor in phosphate buffer at pH 6.5

**Table S1:** Evaluation of the accuracy of the proposed method for determination of VID

| Sample number | Conc. taken (µg/ml) | Conc. Found ^a^ (µg/ml) ± SD | % Recovery ± SD |
| --- | --- | --- | --- |
| 1 | 15 | 14.9 ± 0.09 | 99.6 ± 0.6 |
| 2 | 20 | 19.8 ± 0.02 | 99.0 ± 0.1 |
| 3 | 50 | 50.4 ± 0.03 | 100.8 ± 0.05 |
| 4 | 80 | 81.4 ± 0.12 | 101.75 ± 0.15 |
| 5 | 120 | 119.02 ± 0.28 | 99.18 ± 0.23 |

**^a^** Mean of three replicate measurements, SD: standard deviation.

**Table S2:** Precision data for the determination of VID in pure form by the proposed DPV method.

| Precision level | taken Con.  (µg/ml) | Found ^a^  (µg/ml) ± SD | % Recovery | %RSD |
| --- | --- | --- | --- | --- |
| Intra-day | 50 | 50.13 ± 0.44 | 100.26 | 0.87 |
|  | 80 | 80.4 ± 0.2 | 100.5 | 0.24 |
|  | 120 | 121.7 ± 1.8 | 101.47 | 1.79 |
|  |  |  |  |  |
| Inter-day | 50 | 49.7 ± 0.51 | 99.4 | 1.01 |
|  | 80 | 80.6 ± 0.71 | 100.8 | 0.87 |
|  | 120 | 121.8 ± 1.95 | 101.5 | 1.6 |

**^a^** Mean of three replicate measurements, RSD: relative standard deviation

**Table S3:** Peak current values obtained from five independently prepared modified electrodes (ZNO-NPs/GOs/GCE) using 80 µg/ml vildagliptin

| **Electrode No.** | **di [µA]** |
| --- | --- |
| 1 | 20.39 |
| 2 | 21.53 |
| 3 | 21.15 |
| 4 | 21.21 |
| 5 | 21.35 |
| Mean | 21.13 |
| SD | 0.42 |
| %RSD | 1.99 |

**Table S4:** Application of the proposed DPV method for assay of VID in spiked human plasma.

| **Parameter** | **Conc. Added**  **(µg/ml)** | **Conc. Found^a^**  **(µg/ml)** ± SD | **% Recovery** ± **SD** |
| --- | --- | --- | --- |
| Spiked human plasma | 15 | 15.09 ± 0.15 | 100.6 ± 1.00 |
|  | 20 | 19.8 ± 0.1 | 99 ± 0.49 |
|  | 50 | 49.19 ± 0.3 | 98.38 ± 0.6 |
|  | 80 | 81.35 ± 0.24 | 101.68 ± 0.3 |
|  | 120 | 121.7 ± 0.47 | 101.4 ± 0.39 |

^a^ Mean of five determinations, SD: standard deviation
